# *In situ* synthesis of sub-nanometer metal particles on hierarchically porous metal–organic frameworks *via* interfacial control for highly efficient catalysis†
†Electronic supplementary information (ESI) available: Details of the experimental procedures and other figures and tables. See DOI: 10.1039/c7sc04269h


**DOI:** 10.1039/c7sc04269h

**Published:** 2017-12-14

**Authors:** Pei Zhang, Chunjun Chen, Xinchen Kang, Lujun Zhang, Congyi Wu, Jianling Zhang, Buxing Han

**Affiliations:** a Beijing National Laboratory for Molecular Sciences , Key Laboratory of Colloid and Interface and Thermodynamics , Institute of Chemistry , Chinese Academy of Sciences , Beijing 100190 , P. R. China . Email: hanbx@iccas.ac.cn; b University of Chinese Academy of Sciences , Beijing 100049 , P. R. China

## Abstract

In this work, we developed a strategy to *in situ* synthesize sub-nanometer metal particle/hierarchically mesoporous metal–organic framework (MOF) composites in emulsion. In this route, water droplets in the emulsion acted as both a solvent of the metal precursors and a template for the hierarchical mesopores of MOFs, and the surfactant was an emulsifier and a reductant for generating metal particles. Au/Zn-MOFs (MOFs formed by Zn^2+^ and methylimidazole), Ru/Zn-MOFs, Pd/Zn-MOFs, and Au/Cu-MOFs (MOFs formed by Cu^2+^ and methylimidazole) were prepared using this method, in which ultrafine metal particles (*e.g.* 0.8 nm) were immobilized uniformly on hierarchically mesoporous MOFs. Au/Zn-MOFs and Au/Cu-MOFs showed outstanding catalytic performances for the selective aerobic oxidation of cyclohexene to 2-cyclohexen-1-one in the absence of an initiator, and Ru/Zn-MOFs were very active and selective for the hydrogenation of diphenyl sulfoxide to diphenyl sulfide. In addition, the catalysts were also very stable in the reactions.

## Abstract 2

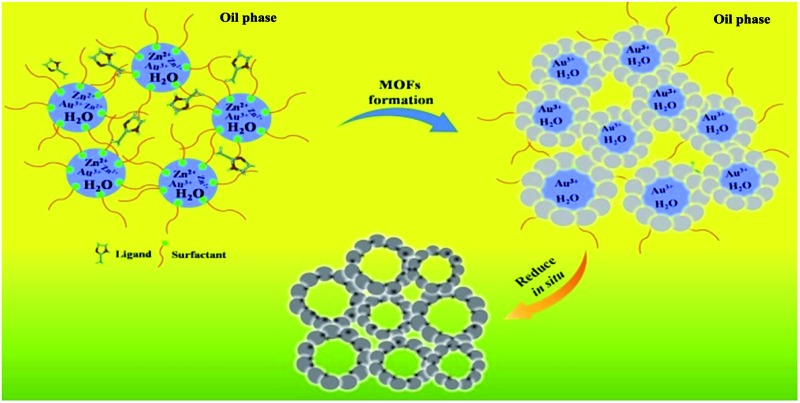
Sub-nanometer metal particle/hierarchically mesoporous metal–organic framework composites can be synthesized *in situ* in bio-based surfactant emulsion.

## Introduction

Metal–organic frameworks (MOFs) represent an emerging class of crystalline materials with diverse and uniform porosity, which are very promising for applications in heterogeneous catalysis.^1^–^3^ Hierarchically porous supports are particularly desired in many catalytic processes because they integrate the advantages of pores with different sizes in the diffusion and adsorption of the species in reaction systems.^4^–^7^

Emulsions are made of liquids, which can dissolve both polar and non-polar substances. The dispersed droplets in emulsions are both highly deformable and easily removable after the accomplishment of templating. Emulsions offer advantages for preparing a wide variety of materials with controlled particle size, morphology and composition at mild conditions.^8^–^11^ Utilization of emulsions as heterogeneous media is a way to obtain porous materials with hierarchical porosity and variable structures.

Sub-nanometer metal particles have unique electronic structures, surface geometric effects, and intrinsic chemical properties.^12^–^14^ For instance, it has been reported that Au particles smaller than 2 nm were effective for catalyzing oxidation reactions and exhibited size-sensitive catalytic properties.^15^,^16^ Controlling the size of metal particles is very difficult because aggregation readily occurs due to high cohesive energy and low melting points.^17^,^18^ Uniform immobilization of sub-nanometer metal particles on hierarchically porous MOFs is very interesting in different fields, but still remains a major technical challenge.

In this work, we found that the bio-based surfactant sorbitol-alkylamine (SAAS-C_12_, Scheme S1†)^19^ could form a stable W/O emulsion. A method to synthesize sub-nanometer metal particle/hierarchically mesoporous MOF composites was proposed using water droplets in the emulsion as a template for the hierarchical pores and a solvent of the precursors, and the surfactant acted as both an emulsifier and a reductant *in situ*. Au/Zn-MOFs (MOFs formed by Zn^2+^ and methylimidazole), Ru/Zn-MOFs, Pd/Zn-MOFs, and Au/Cu-MOFs were prepared using this method, in which the ultrafine metal particles (*e.g.* 0.8 nm) were immobilized uniformly on hierarchically mesoporous MOFs. The catalysts demonstrated outstanding catalytic performances because they integrated the advantages of the ultrafine metal particle catalysts and the hierarchically porous supports.

## Results and discussion

**Scheme 1:**
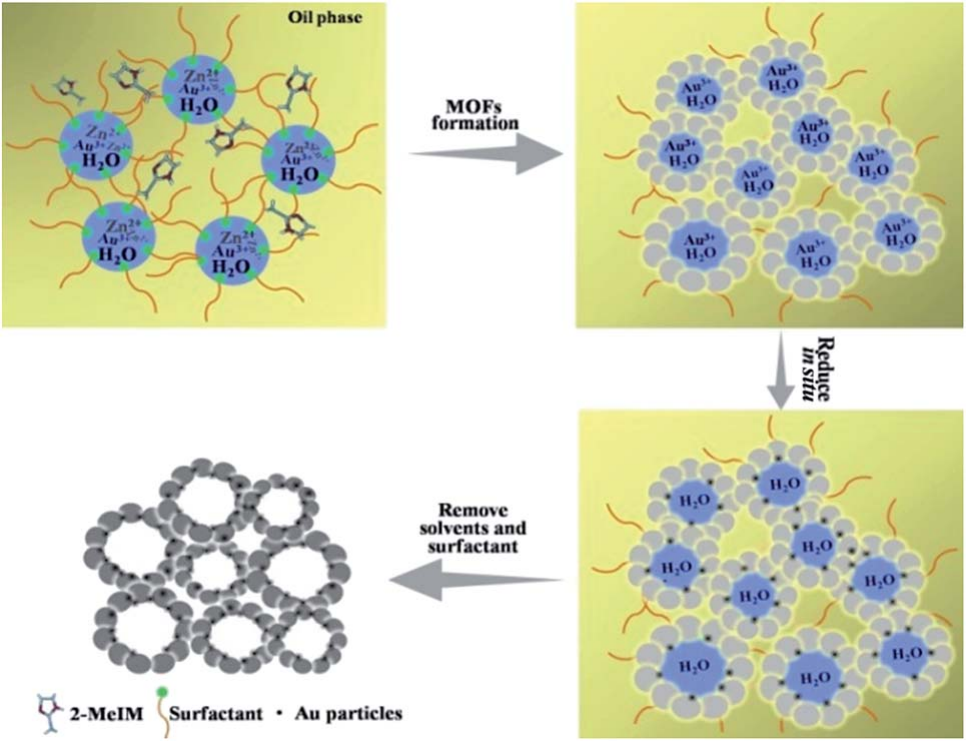
Schematic illustration of the method for *in situ* preparation of Au/Zn-MOFs in emulsion.

The formation of the emulsion was confirmed by fluorescence microscopic analysis with water soluble dye (Rhodamine B), and the size distribution of the water droplets in the emulsion was determined using dynamic light scattering (DLS). The sizes of water droplets ranged from about 5 nm to 60 nm. The results are discussed in detail in the ESI (Fig. S1 and the corresponding discussion†). Taking the synthesis of Au/Zn-MOFs as the example, the proposed method to prepare metal particle/MOF composites is illustrated in Scheme 1, and the details are given in the method section. In the preparation, Zn^2+^ and Au^3+^ were dissolved in water droplets, and the ligand methylimidazole was in the oil phase. Zn-MOFs were firstly formed at water/oil interfaces surrounding the water droplets which acted as templates for the mesopores. Then Au^3+^ ions were reduced *in situ* after heat treatment by the secondary amine and hydroxy groups in the surfactant, which both acted as an emulsifier and reductant.^20^,^21^ Therefore, the Au^3+^ ions were reduced by the surfactant *in situ* after heating from 25 °C to 35 °C, and the yielded Au particles were immobilized on the wall of the porous MOFs. Au/Zn-MOF composites were obtained after removing the solvents. It is known that the characteristics of the pores of the composites are closely related with the size of the droplets (Fig. S1†). In addition, hierarchically porous MOFs without metal particles could also be prepared in the absence of Au^3+^ (Fig. S2†).

**Fig. 1:**
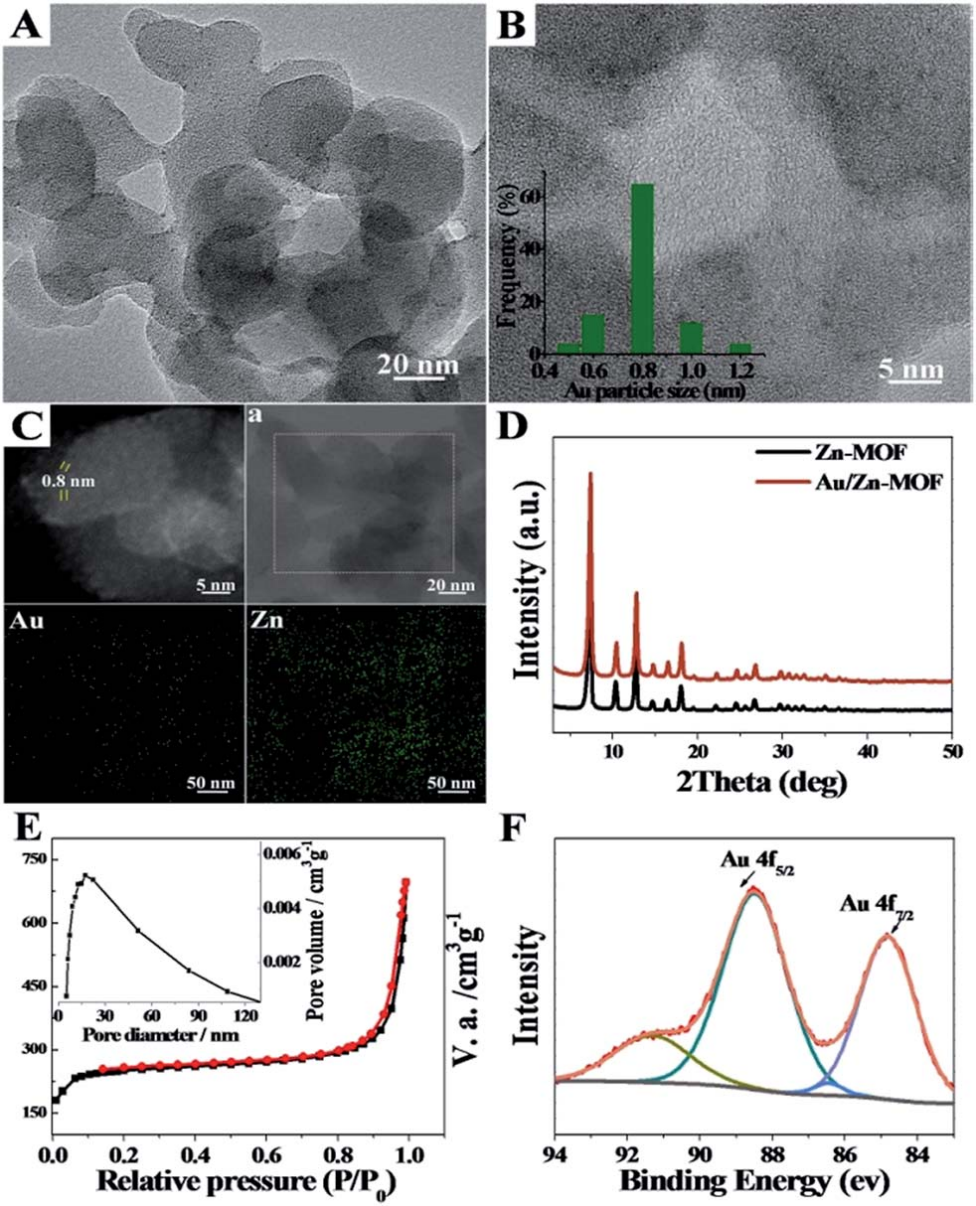
Structural characterizations of the Au/Zn-MOF. (A) Representative TEM image of Au/Zn-MOF; (B) high-resolution TEM (HRTEM) image and the lower inset is the particle size distribution of Au particles; (C) representative HAADF-STEM image and STEM-EDX elemental mapping of the Au/Zn-MOF, the selected area is framed in (a); (D) XRD patterns of the obtained Au/Zn-MOF (b) and Zn-MOF (a); (E) N_2_ adsorption/desorption isotherms and the mesopore size distribution (inset); (F) XPS spectra of Au 4f; Au loading was 0.8 wt% determined by ICP-AES.

The transmission electron microscopy (TEM) image of the Au/Zn-MOF is shown in Fig. 1A. The HRTEM and high-angle annular dark-field HAADF images with high magnification showed that sub-nanometer Au particles (0.8 nm) were decorated uniformly on the hierarchically porous Zn-MOF (Fig. 1B and C). The corresponding energy dispersive X-ray spectroscopy (STEM-EDX) elemental mapping analysis (Fig. 1C) also revealed that Au particles were evenly immobilized on the supports. The powder X-ray diffraction (PXRD) pattern of the obtained Zn-MOF was the same as that reported,^22^ and patterns of the Zn-MOF and Au/Zn-MOF were nearly the same (Fig. 1D). In the XRD pattern of the Au/Zn-MOF, the diffraction peaks for Au particles were very weak due to their small size. The porosity of the Au/Zn-MOF was characterized by N_2_ adsorption/desorption measurements. The BET surface area, total pore volume, and average pore diameter are provided in Table S1.† The size distribution of the mesopores of the Au/Zn-MOF was similar to that of the water droplets in the emulsion (Fig. 1E and S1†), indicating the template function of the water droplets. The X-ray photoelectron spectroscopy (XPS) analysis of Au (Fig. 1F) showed two pairs of spin–orbit doublets, indicating the existence of Au^0^ at 84.8 and 88.5 eV and Au^+^ at 86.4 and 91.3 eV. The positive shift in the binding energy of Au 4f_7/2_ confirmed the electronic effect of small particle size.^23^ Furthermore, a shift to the higher binding energy of Au 4f_5/2_ was consistent with the negative shift of the Zn^2+^ spectrum with respect to the origin Zn-MOF (Fig. S3†). Thus, an interaction between Au particles and Zn^2+^ existed in the composites,^24^,^25^ which also indicated the immobilization of Au on the support. In this route, the water droplets in the emulsion acted both as a solvent of the metal precursors (Zn^2+^ and Au^3+^) and a template for the mesopores of Zn-MOF. The surfactant behaved as an emulsifier and reducing agent of Au^3+^. Zn-MOFs were firstly formed from Zn^2+^ and the ligand around the water droplets in the emulsion, leaving Au^3+^ ions in the water droplets and isolated by the mesopores of the Zn-MOF. Due to the wide size distribution of the water droplets in the emulsion, hierarchically mesoporous Zn-MOF was formed. After Au^3+^ ions were reduced *in situ*, the Au particles were immobilized uniformly on the Zn-MOF. For comparison, the experiment was conducted by adding HAuCl_4_ solution after the formation of Zn-MOF. Clearly, the agglomeration of Au particles occurred as shown in Fig. S4,† further showing the advantage of the *in situ* method proposed in this work. In addition, the size of Au particles could be tuned by the concentration of Au precursor using the procedures, and Au/Zn-MOF composites with average Au particle sizes of 0.8 nm, 1.0 nm, 1.5 nm and 2.0 nm were obtained (Fig. 1 and S5†).

**Fig. 2:**
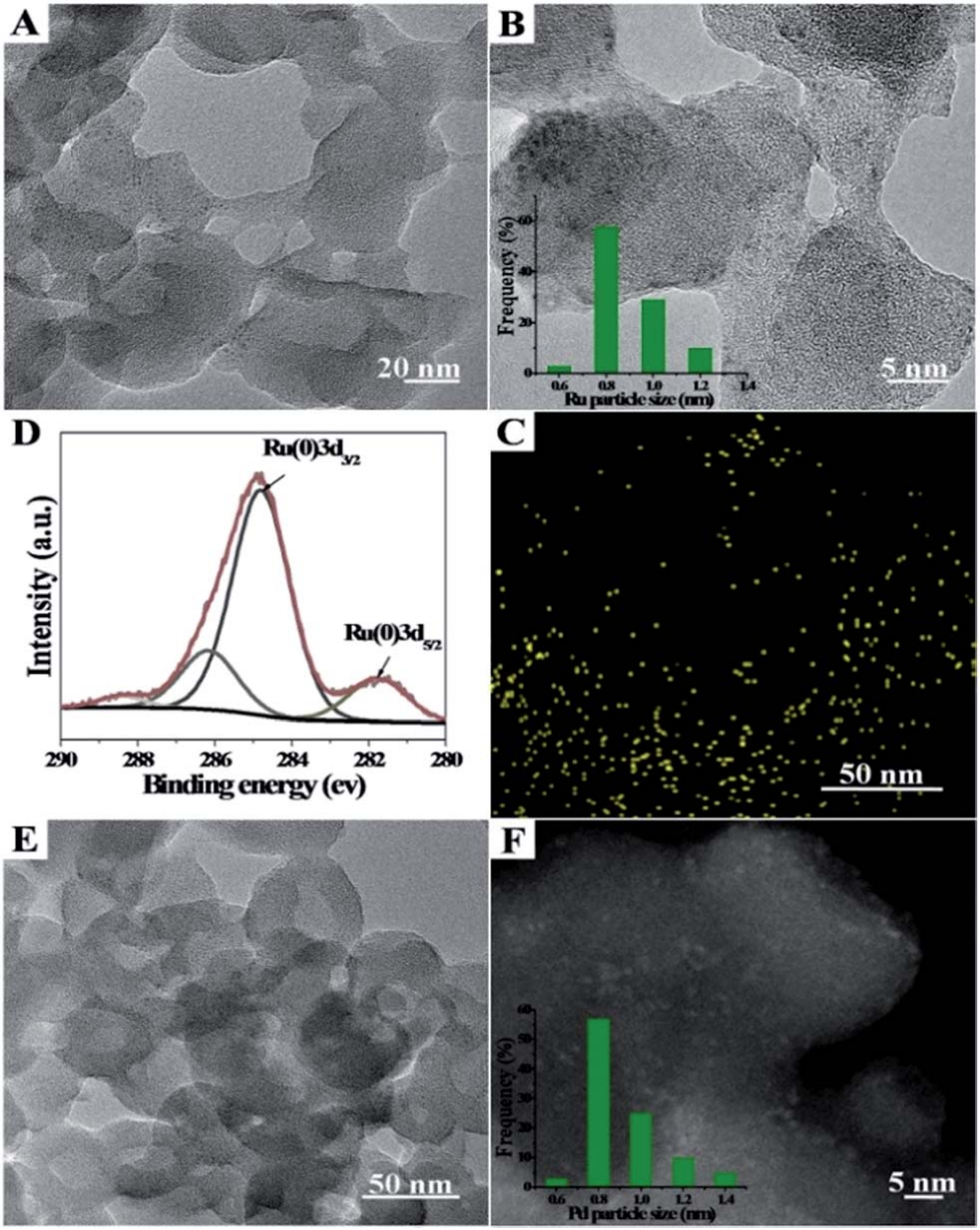
Characterizations of Ru/Zn-MOF and Pd/Zn-MOF composites. (A) A representative TEM image of the Ru/Zn-MOF; (B) HRTEM image and the lower inset is the particle size distribution of Ru particles; (C) STEM-EDX elemental mapping of Ru/Zn-MOF and Ru elements; the selected area is framed in (A); (D) XPS spectra of Ru 3d and (E and F) a representative TEM image and HAADF-STEM image of Pd/Zn-MOFs. Ru loading in Ru/ZIF-8 was 0.6 wt% and Pd loading in Pd/Zn-MOF was 2.3 wt% as determined by ICP-AES.

Ru/Zn-MOFs and Pd/Zn-MOFs were also prepared by this method using RuCl_3_ and Pd(NO_3_)_2_ as metal precursors, respectively. The characterizations showed that Ru or Pd particles of 0.8 nm could also be supported on hierarchically porous Zn-MOFs uniformly (Fig. 2). These composites were characterized by TEM, XPS, XRD and N_2_ adsorption/desorption methods, and the corresponding results are provided in Fig. S6 and S7.† The BET surface area, total pore volume, and average pore diameters of the composites are listed in Table S1.†

The size of metal particles is a crucial factor for catalytic performance in heterogeneous catalysis.^26^ It has been shown that Au nanoparticles of a small size could selectively catalyze oxidation of alkenes, which is a very important reaction.^27^,^28^ However, performing the oxidation of alkenes without the addition of an initiator both selectively and efficiently remains a challenge.^29^ We studied the catalytic performances of the Au/Zn-MOF composites with different Au particle sizes for aerobic oxidation of cyclohexene to 2-cyclohexen-1-one in the absence of an initiator, and the results are given in Table 1. The catalyst with a Au size of 0.8 nm had outstanding activity and selectivity. Au/Zn-MOF catalysts with larger Au particles showed lower activity and selectivity. The stability of the catalyst with a Au size of 0.8 nm was studied, and the activity and selectivity did not change after being reused four times (Fig. S8a†). The TEM and XRD characterizations indicated that the morphology and structure of the catalyst did not change obviously after four cycles (Fig. S8b and c†), further indicating the excellent stability of the catalyst.

**Table 1:** The conversion and selectivity of cyclohexene oxidation over Au/Zn-MOF catalysts with different Au particle sizes^*a*^

Entry	Au size (nm)	*C* (%)	Selectivity (%)		
			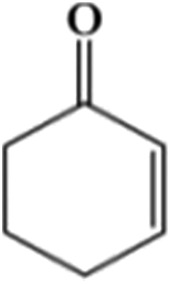	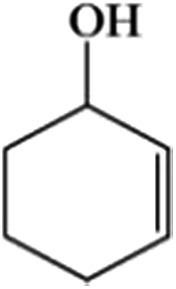	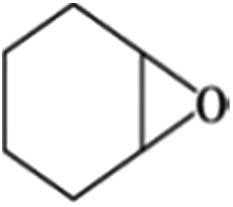
Au/Zn-MOF^*b*^	0.8	82	98	—	—
Au/Zn-MOF^*c*^	1.0	79	95	<2	—
Au/Zn-MOF^*d*^	1.5	64	81	11	<3
Au/Zn-MOF^*e*^	2.0	34	66	21	—
Au/Cu-MOF^*f*^	0.8	77	97	—	<1
Zn-MOF	—	7.4	23	19	14

^*a*^Reaction conditions: 30 mg catalyst, 0.5 mmol cyclohexene, 2 ml dioxane, 100 °C, 1 MPa O_2_ and 8 h.

^*b*^Au loading 0.8 wt%.

^*c*^Au loading 1.1 wt%.

^*d*^Au loading 1.3 wt%.

^*e*^Au loading 2.0%.

^*f*^Au loading 0.6 wt%; metal loadings were determined by ICP-AES; the conversion and selectivity were determined by gas chromatography, and the conversion was defined based on the initial cyclohexene present. *C* = conversion.

To further confirm that ultrafine Au particles were crucial for the high activity and selectivity, Au particles with a size of 0.8 nm on the Cu-MOF were also prepared by this route, and the characterizations are provided in Fig. S9.† The activity and selectivity of the reaction over Au/Cu-MOFs were also very high as shown in Table 1.

**Fig. 3:**
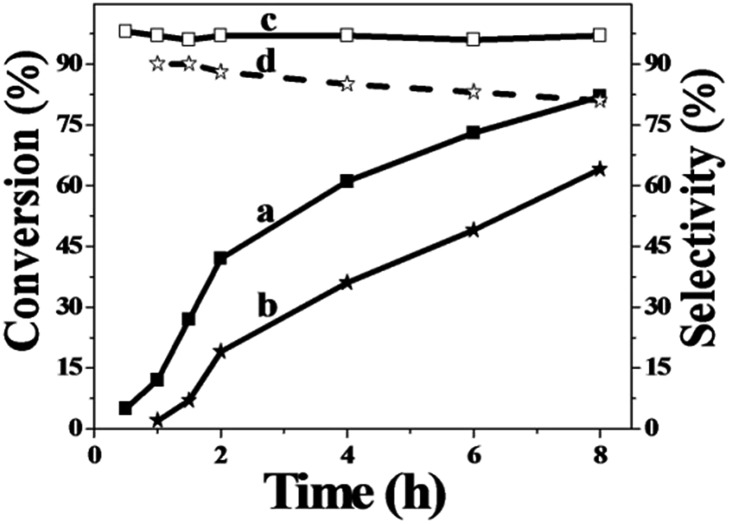
Dependence of cyclohexene conversion and 2-cyclohexene-1-one selectivity on the reaction time over Au/Zn-MOF catalysts at the reaction conditions of Table 1. (a and c) Conversion and selectivity with 0.8 nm Au particles and (b and d) conversion and selectivity with 1.5 nm Au particles.

In order to study the catalytic nature of Au particles, reaction profiles with time over Au/Zn-MOFs with Au particles of 0.8 nm and 1.5 nm were investigated, and the results are given in Fig. 3. It can be observed that the conversion and selectivity of the reaction over the catalyst with smaller Au particles (0.8 nm) were higher at all reaction times. The mechanism of the reaction has been well studied.^30^,^31^ In the reaction, cyclohexene was oxidized to form hydroperoxide, which can be further transformed into 2-cyclohexen-1-one immediately, or react with a substrate to form cyclohexen-1-ol and epoxide. It can be deduced from the results in Fig. 3 that both reaction rates from cyclohexene to intermediate hydroperoxide and further to 2-cyclohexen-1-one over the smaller Au particles were higher, yielding higher conversion and selectivity.

The oxidation and reduction of sulfur-based compounds have been studied widely.^32^ However, the design of highly efficient catalysts is still desirable. It was reported that heterogeneous Ru nanoparticle catalysts could accelerate the hydrogenation of sulfoxides to sulfides.^33^ Herein, we studied the catalytic performance of the obtained Ru/Zn-MOF with a particle size of 0.8 nm, and the results are presented in Table 2. The activity of the Ru/Zn-MOF was much higher than that of the commercial Ru/C catalyst with a Ru particle size of 2.5 nm (characterization in Fig. S10†) and that of the reported Ru/TiO_2_ catalyst with a Ru particle size of 1.6 nm.^33^ Moreover, the reusability of the Ru/Zn-MOF with a Ru particle size of 0.8 nm was checked, which showed no change in the activity after recycling 5 times (Fig. S11a†). The morphology and XRD pattern of the used catalyst were not changed (Fig. S11b and c†), showing the excellent stability of the catalyst.
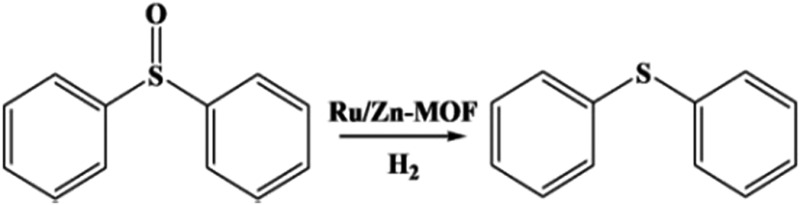


**Table 2:** Catalytic performances of the Ru/Zn-MOF for the hydrogenation of diphenyl sulfoxide^*a*^

Entry	Ru size (nm)	*T* (°C)	*C* ^*b*^	*Y* ^*c*^	TOF^*d*^ (h^–1^)
1^*e*^	0.8	95	>99	98	207.6
2^*f*^	2.5	95	21	19	2.4
3^*g*^	1.6	100	>99	>99	20.8

^*a*^Reaction conditions: catalyst (40 mg), diphenyl sulfoxide (0.5 mmol), 1,4-dioxane (2 ml), 5 atm H_2_ and 1 h.

^*b*^Determined by GC using isopropanol as an internal standard.

^*c*^Determined by GC using isopropanol as an internal standard.

^*d*^TOF denotes moles of sulfide per mole of Ru per hour; the Ru loading was 0.6 wt% as determined by ICP-AES.

^*e*^Ru/Zn-MOF catalysts synthesized by the route proposed herein.

^*f*^Commercial Ru/C catalyst with 5 wt% Ru loading.

^*g*^ Ref. 17; *C* = conversion%; *Y* = yield%.

## Conclusions

In conclusion, Au/Zn-MOFs, Au/Cu-MOFs, Ru/Zn-MOFs, and Pd/Zn-MOFs with sub-nanometer metal particles were *in situ* synthesized in emulsion using the surfactant SAAS-C_12_ as an emulsifier and reductant. In this route, metal precursors of MOFs (*e.g.* Zn^2+^) and metal particles (*e.g.* Au^3+^) were dissolved in water droplets, and the ligand existed in the oil phase. MOFs were firstly formed around the water droplets to produce a hierarchically mesoporous structure. Then, the metal precursors isolated in the mesopores were reduced *in situ* by the surfactant, leaving metal particles immobilized uniformly on the hierarchical pores of MOFs. In addition, the size of the metal particles could be easily controlled by the content of the metal precursors. The Au/MOFs with sub-nanometer Au particles (0.8 nm) had a much higher activity and selectivity for the aerobic oxidation of cyclohexene to 2-cyclohexen-1-one than those with larger Au particles, and the Ru/Zn-MOFs with Ru particles of 0.8 nm were much more effective than those with larger Ru particles as a catalyst for the hydrogenation of diphenyl sulfoxide to diphenyl sulfide. We believe that this simple one-step method can also be used to synthesize some other supported metal catalysts, in which sub-nanometer metal particles are decorated uniformly on hierarchically mesoporous MOFs.

## Conflicts of interest

There are no conflicts to declare.

## Supplementary Material

Supplementary information
