# Supplementary material for: In situ synthesis of sub-nanometer metal particles on hierarchically porous metal–organic frameworks via interfacial control for highly efficient catalysis
Source: Chem Sci. 2017 Dec 14;9(5):1339–43. doi: 10.1039/c7sc04269h (PMC5887229; doi:10.1039/c7sc04269h)
Supplement: Supplementary file 1 [file SC-009-C7SC04269H-s001.pdf]

Electronic Supplementary Material (ESI) for Chemical Science.
This journal is © The Royal Society of Chemistry 2017

In situ Synthesis of Sub-nanometer Metal Particles on Hierarchically Porous Metal-Organic Frameworks via Interfacial Control for Highly Efficient Catalysis

Pei Zhang,[a,b] Chunjun Chen,[a,b] Xinchen Kang,[a,b] Lujun Zhang,[a,b] Congyi Wu,[a] Jianling Zhang,[a,b] and Buxing Han*[a,b]

a Beijing National Laboratory for Molecular Sciences, Key Laboratory of Colloid and Interface and Thermodynamics, Institute of Chemistry, Chinese Academy of Sciences, Beijing 100190, China

b University of Chinese Academy of Sciences, Beijing 100049, China

E-mail: hanbx@iccas.ac.cn

This file includes:

Experimental Section

Supplementary Structure: Scheme S1

Supplementary Figures: FigureS1-S11

Supplementary Table: Table S1

Experimental Section

Materials: Zinc chloride (98%), 2-methylimidazole (99%, 2-MeIM), $RuCl_3·3H_2O$ (Ru ≥ 38%), $CuCl_2·2H_2O$ (99%), cyclohexene (99%), 2-cyclohexen-1-one (98%) and phenyl sulfide (99%) were purchased from Sigma-Aldrich. 2-Cyclohexen-1-ol was purchased from Tokyo Chemical Industry. Co., Ltd. *cis*-1,2-Cyclooctanediol (95%), $Pd(NO_3)_2·nH_2O$ (Pd ⩾ 39%) and diphenyl sulfoxide (98+%) were obtained from Alfa

Aesar. $HAuCl_4·4H_2O$ (Au ≥ 47.8%), NaOH (A. R. grade), K_2CO_3 (A. R. grade), dichloromethane (A. R. grade), acetone (A. R. grade), isopropanol (A. R. grade), and methanol (A. R. grade) were obtained from Sinopharm Chemical Reagent Co., Ltd. H_2 (> 99.99%), and O_2 (> 99.95%) were supplied by Beijing Analytical Instrument Company. The commercial Ru/C catalyst (5 wt% Ru) was purchased from Baoji Ruike Corporation, China. The surfactant sorbitol-alkylamine (SAAS-C_{12}) was synthesized in our group,[1] and the structure is shown in Scheme S1.

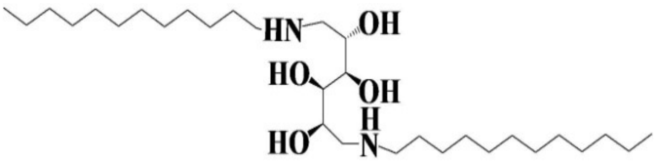

Scheme S1. The structure of bio-based surfactant sorbitol-alkylamine SAAS-C_{12}.

Emulsion preparation and characterizations: To prepare the emulsion, 0.1 mmol (60 mg) of SAAS-C_{12} was added into a glass bottle, and dichloromethane (72 ml) and water (16 ml) were added. The mixture was stirred rapidly at room temperature (25 °C) for 5 min. Then water-in-oil (W/O) emulsion was obtained. The emulsion was characterized by light microscopy and dynamic light scattering (DLS), which are commonly used method.[2]

The microscopy study of emulsions was conducted using a microscope (olympus, IX83, Japan) using Rhodamine B. For fluorescence microscopic analysis, water was stained with 5 µl Rhodamine B (0.1%, w/v), and the emulsion preparation was then followed as described above. The size distribution of the droplets in emulsion was obtained by using dynamic light scattering (DLS). Measurements were carried out using an LLS spectrometer (ALV/SP-125) with a multi-τ digital time correlater (ALV−5000). Light of λ = 632.8 nm from a solid-state He-Ne laser (22 mW) was used as the incident beam. The measurements were conducted at a scattering angle of 90°. The correlation function was analyzed from the scattering data via the CONTIN method to obtain the distribution of diffusion coefficients (D) of the solutes. All the measurements were performed at 25.0 (± 0.1 °C).

Synthesis of Metal-Organic Frameworks (MOFs) in the W/O Emulsion: The method to synthesize MOFs were similar to that reported by other authors.[3] The main difference was that the MOFs were synthesized in emulsion. As example, preparation of the MOF formed by Zn^{2+} and 2-MeIM (Zn-MOF) was discussed. 68 mg of $ZnCl_2$ was dissolved into 16 ml of water containing 60 mg surfactant and 62 ml of dichloromethane, stirring rapidly for 5 min to form emulsion. After that, 122 mg of 2-MeIM in 10 ml dichloromethane solution was added into the emulsion and stirred at

room temperature for 8 h. Then, the obtained solid sample (white powders) was washed with ethanol/water alternately for five times and dried in the vacuum oven at 70 ^oC for 12 h.

***In Situ* Preparation of Au/Zn-MOF, Au/Cu-MOF, Ru/Zn-MOF, and Pd/Zn-MOF.** The synthesis process was similar to that for preparation of the corresponding MOFs discussed above. The main differences were that various metal precursor $HAuCl_4$/$RuCl_3·3H_2O$ or $Pd(NO_3)_2·nH_2O$ was added into the aqueous phase respectively during the emulsification process. We described the procedures to synthesize Au/Zn-MOF in detail because that the synthesis of Au/Cu-MOF, Ru/Zn-MOF and Pd/Zn-MOF were similar. In a typical experiment to prepare Au/Zn-MOF (0.8 wt% Au loading) with 0.8 nm Au particles, 0.5 mmol (68 mg) $ZnCl_2$ was dissolved into 14 ml water, and mixed with 2 ml 6 mmol/L $HAuCl_4$ aqueous solution (pH = 7.0, adjusted by NaOH freshly). Subsequently 62 ml dichloromethane solution containing 60 mg SAAS-C_{12} was mixed to form emulsion with stirring rapidly for 5 min, after that another 10 ml dichloromethane solution containing 1.5 mmol ligand 2-MeIM (122 mg) was added into the emulsion. Firstly, Zn-MOF was synthesized at water droplets/dichloromethane interfaces at 25 ^oC for 7 h, then the emulsion was heated to 35 °C for 30 min for reducing Au^{3+}. The obtained Au/Zn-MOF precipitates (pale yellow powder) were separated, washed with water/acetone alternately for 5 times, and then dried under vacuum at 70 °C for 12 h.

For comparison, Au/Zn-MOF composite was prepared using two-step method by adding $HAuCl_4$ solution (Au precursor) after Zn-MOF formation. The Zn-MOF was first synthesized in the emulsion at room temperature using the similar method discussed above. The only difference was that Au precursor was not added. After the formation of Zn-MOF in the emulsion, 2 ml of 6 mmol/L $HAuCl_4$ aqueous solution (pH = 7.0, adjusted by NaOH) was added to the emulsion dropwise. The emulsion was stirred at 25 ^oC for another 5 h and heated to 35 °C for 30 min to reduce Au^{3+}. The obtained Au/Zn-MOF precipitates (pale yellow powder) were separated, washed with distilled water/acetone alternately for 5 times, and then dried under vacuum at 70 °C for 12 h.

To prepare Au/Zn-MOF with 1.0 nm Au particles (1.1 wt% Au loading), Au/Zn-MOF with 1.5 nm Au particles (1.3 wt% Au loading), and Au/Zn-MOF with 2.0 nm Au particles (2.0 wt% Au loading), 2 ml of 16 mmol/L, 2 ml of 25 mmol/L, and 3 ml of 30 mmol/L $HAuCl_4$ aqueous solutions were used (pH = 7.0, adjusted by NaOH freshly), and other conditions were the same as that for preparing Au/Zn-MOF with 0.8 nm Au particles. The procedures to synthesize Au/Cu-MOF with 0.8 wt% and 0.8 nm Au particles was similar to that for preparing Au/Zn-MOF with 0.8 nm Au particles. The different was that 0.5 mmol (85 mg) $CuCl_2·2H_2O$ was used to replace $ZnCl_2$. For

preparing the Ru/Zn-MOF and Pd/Zn-MOF, 2 ml 5 mmol/L $RuCl_3 \cdot 3H_2O$ aqueous solution and 3 ml 30 mmol/L $Pd(NO_3)_2 \cdot nH_2O$ aqueous solutions were used, respectively.

We would like to mention a phenomenon that if the oxidation ability of the metal ions is very strong, the metal ions will be reduced spontaneously before MOF generation, and subnanometer particles were not easy to obtain in this method.

Light Microscopy. The microscopy study of emulsions was conducted using a microscope (olympus, IX83, Japan). For fluorescence microscopic analysis, water was stained with 5 μL Rhodamine B (0.1%, w/v), and the emulsion preparation was then followed as described above.

Droplet Size Distribution of the Emulsion. The size distribution of the droplets in emulsion was obtained by using dynamic light scattering (DLS). Measurements were carried out using an LLS spectrometer (ALV/SP-125) with a multi-τ digital time correlater (ALV−5000). Light of λ = 632.8 nm from a solid-state He-Ne laser (22 mW) was used as the incident beam. The measurements were conducted at a scattering angle of 90°. The correlation function was analyzed from the scattering data via the CONTIN method to obtain the distribution of diffusion coefficients (D) of the solutes. All the measurements were performed at 25.0 (± 0.1 °C).

Material Characterizations: Powder XRD analysis of the samples was performed on the X-ray diffractometer (Model D/MAX2500, Rigaka) with Cu-Kα radiation, and the scan speed was 5°/min. The morphologies of Zn-MOF, Cu-MOF, Au/Zn-MOF, Au/Cu-MOF, Ru/Zn-MOF and Pd/Zn-MOF were characterized by scanning electron microscope (SEM) (TECNAI 20PHILIPS electron microscope) and transmission electron microscope (TEM) (JEOL-1011 and JEOL-2100F). HAADF images were obtained by JEOL-2100F. The porosity properties of the materials were determined by N_2 adsorption-desorption isotherms using a Micromeritics ASAP 2020M system. The Au, Ru and Pd loadings in the catalysts were determined by ICP-AES method. X-ray photoelectron spectroscopy (XPS) analysis was performed on the Thermo Scientific ESCALab 250Xi using a 200 W monochromated Al Kα radiation.

Typical procedure for the catalytic oxidation of cyclohexene over Au/Zn-MOF: The aerobic oxidation of cyclohexene was conducted in a 20 ml stainless steel batch reactor. In the experiment, 30 mg catalyst, 0.5 mmol cyclohexene, and 2 ml dioxane were added into the reactor. O_2 was introduced into the system at 1 MPa and the pressure was maintained during the reaction. The reactor was placed in oil bath of desired temperature. The reaction mixture was stirred for desired time. Then, the reaction mixture was cooled in ice-water and O_2 was released slowly. The reaction

mixture was analyzed using a gas chromatograph (GC, HP 4890) equipped with a flame ionization detector (FID), and isopropanol used as the internal standard. In the reuse experiments, the catalyst was separated by centrifugation and washed with acetone for four times, then used for the next run after drying at 70 ^{o}C under vacuum.

Typical procedure for the catalytic hydrogenation of diphenyl sulfoxide over Ru/Zn-MOF: The experimental procedure was similar to that discussed above. In the experiment, 40 mg Ru/Zn-MOF was placed into the 20 ml stainless steel batch reactor. 0.5 mmol diphenyl sulfoxide and 2 ml dioxane were added into the reactor. H_2 was introduced into the system at 5 atm and the pressure was maintained during the reaction. The reactor was placed in oil bath of desired temperature. The reaction mixture was cooled in ice-water and the gas was released. The reaction mixture was centrifuged to separation and analyzed using a gas chromatograph (GC, HP 4890) equipped with a flame ionization detector (FID), and isopropanol was used as the internal standard. In the reuse experiments, the catalyst was separated by centrifugation and washed with acetone for four times, then used for the next run after drying at 70 ^{o}C under vacuum.

Results and discussion

1. Characterization of the emulsion formed by the bio-based surfactant (SAAS-C_{12}).

The formation of the emulsion was studied by visual observation and light microscopy. The size distribution of the water droplets in CH_2Cl_2/water emulsion was characterized by light microscopy with water soluble dye (Rhodamine B) and dynamic light scattering (DLS), as showed in Figure S1c. The size of the droplets was in the range of 5 ～ 60 nm.

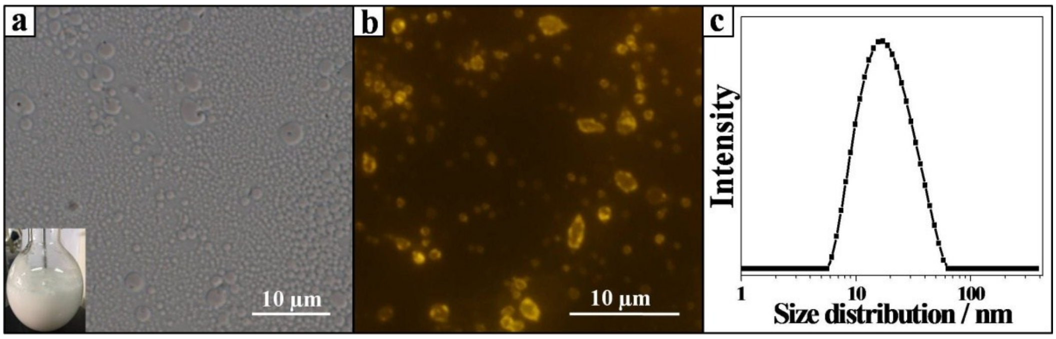

Figure S1. The optical micrographs of the emulsion stabilized by SAAS-C_{12} in CH_2Cl_2/water (a) and the fluorescence microscopic analysis using Rhodamine B (b) respectively; (c) The particle size distribution of water droplets in the emulsion was determined by DLS.

2. The formation of MOFs using emulsion as template.

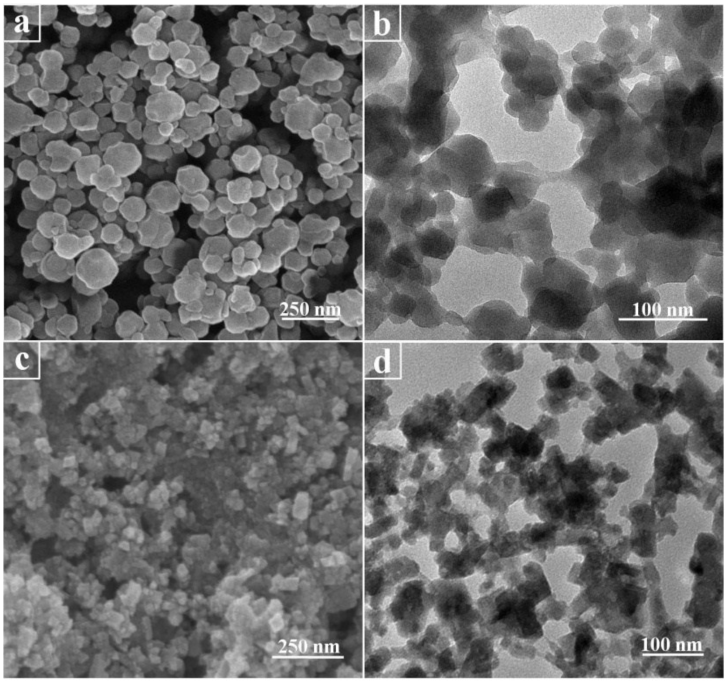

Figure S2. Characterizations of Zn-MOF and Cu-MOF: (a, c) Representative SEM images of Zn-MOF and Cu-MOF; (b, d) Representative TEM images of Zn-MOF and Cu-MOF respectively.

Supplementary Figures

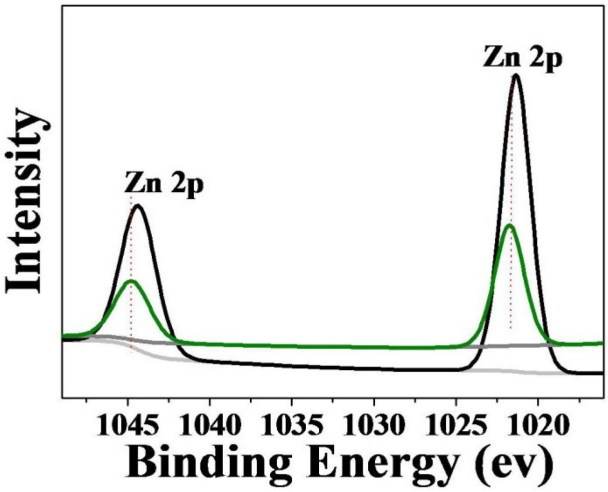

Figure S3. XPS spectra of Zn 2p of Au/Zn-MOF (0.8 wt%) with 0.8 nm Au particles and the corresponding spectrum of Zn-MOF (green curve). A shift to lower binding energy is observed for Zn 2p of Au/Zn-MOF, indicating the presence of interaction between Au and Zn.

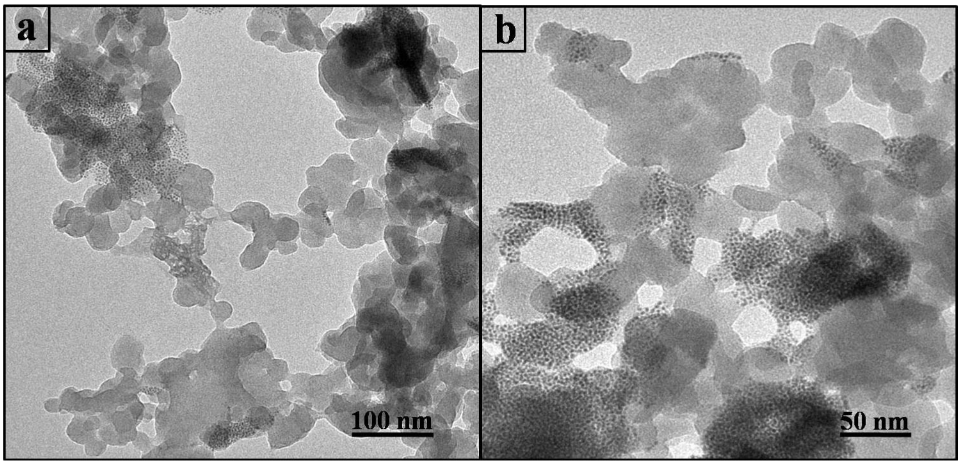

Figure S4. Representative TEM images of Au/Zn-MOF obtained by adding Au precursors after the formation of Zn-MOF.

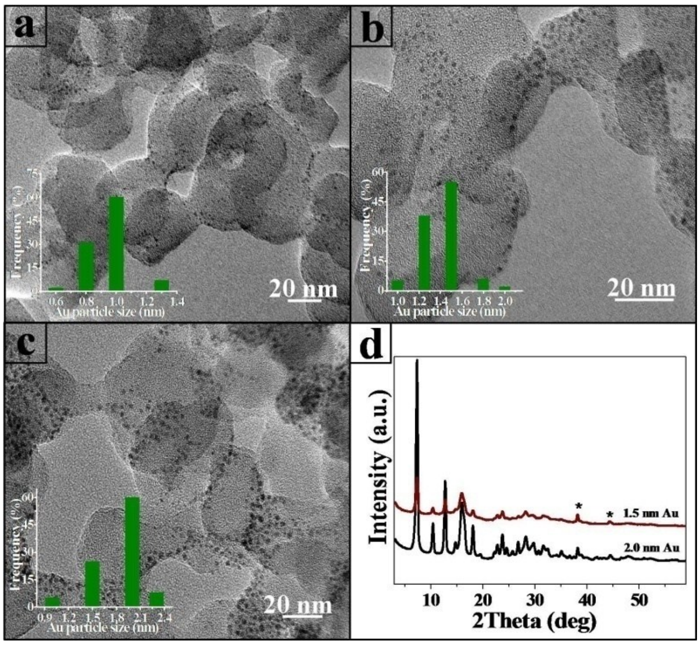

Figure S5. Representative TEM images of Au/Zn-MOF with different Au contents and particle sizes. (a) Au/Zn-MOF with 1.1 wt% Au and 1.0 nm Au particles; (b) Au/Zn-MOF with 1.3 wt% Au and 1.5 nm Au particles; (c) Au/Zn-MOF with 2.0 wt% Au and 2.0 nm Au particles; (d) XRD patterns of Au/Zn-MOF with 1.5 nm and 2.0 nm Au particles.

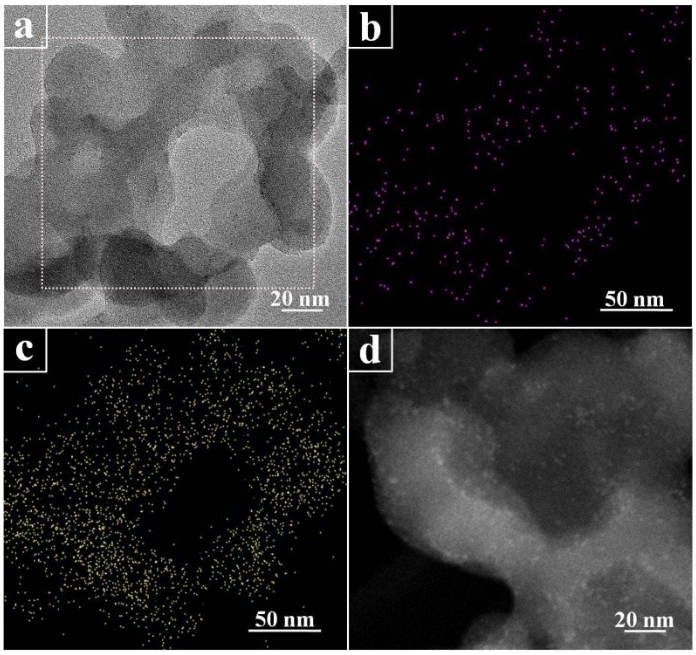

Figure S6. Characterizations of Pd/Zn-MOF. **(**a) Representative TEM images of Pd/Zn-MOF with 2.3 wt% Pd loading determined by ICP-AES; (b, c) STEM-EDX elemental mapping (b) represents Pd elements; c represents Zn elements of the Pd/Zn-MOF, and the area is framed in (a); (d) Representative HAADF-STEM image of the Pd/Zn-MOF.

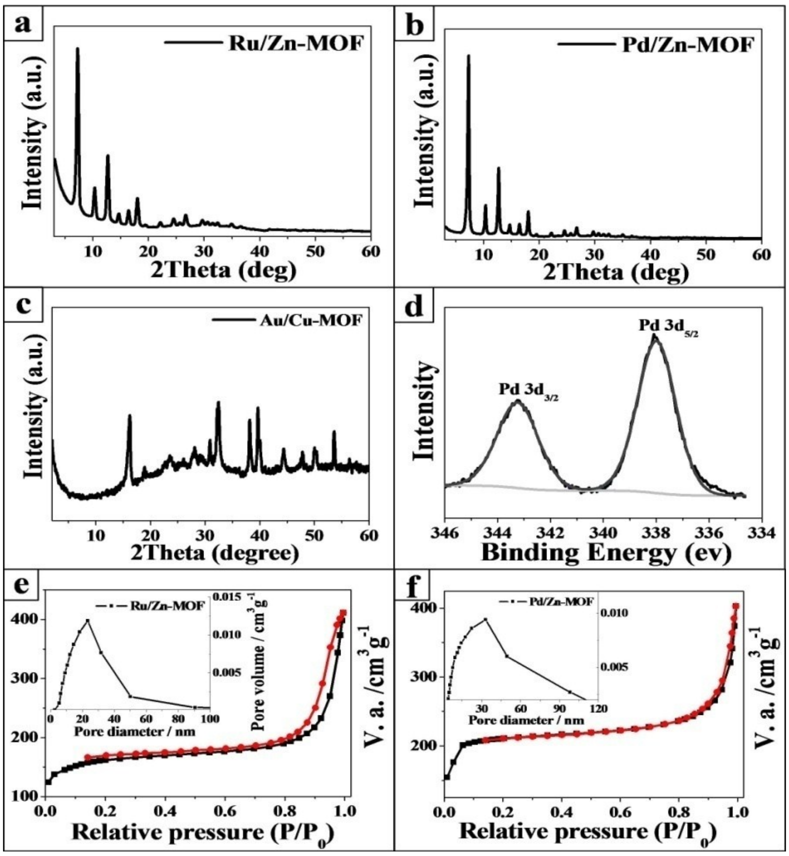

Figure S7. Characterizations of the catalysts: (a) XRD pattern of Ru/Zn-MOF with 0.6 wt% Ru determined by ICP-AES; (b, d) XRD pattern and XPS spectrum of Pd/Zn-MOF with 2.3 wt% Pd determined by ICP-AES; (c) XRD pattern of Au/Cu-MOF with 0.6 wt% Au; (e, f) N_2

adsorption/desorption isotherms and mesopore size distribution (insets) of the Ru/Zn-MOF and Pd/Zn-MOF, respectively.

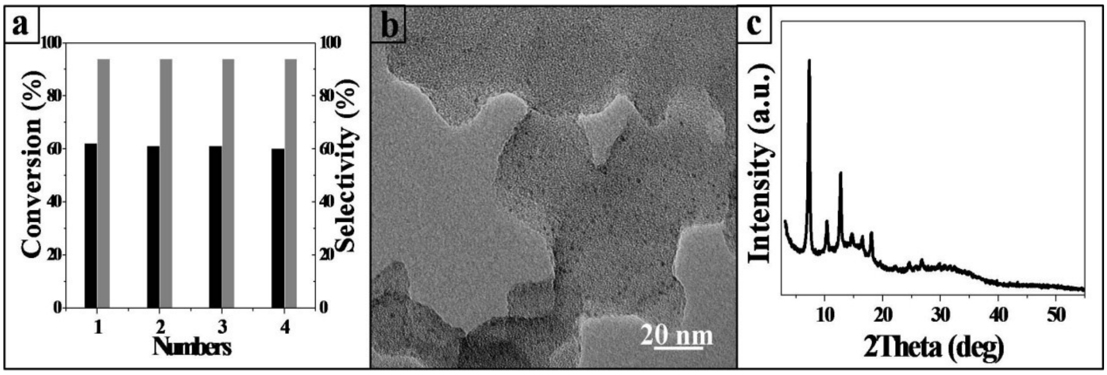

Figure S8. (a) Reusability of Au/Zn-MOF catalyst with reaction time of 4 h and the other reaction conditions were the same as entry 1 of Table 1; TEM image (b) and XRD pattern (c) of Au/Zn-MOF catalyst after used for four times.

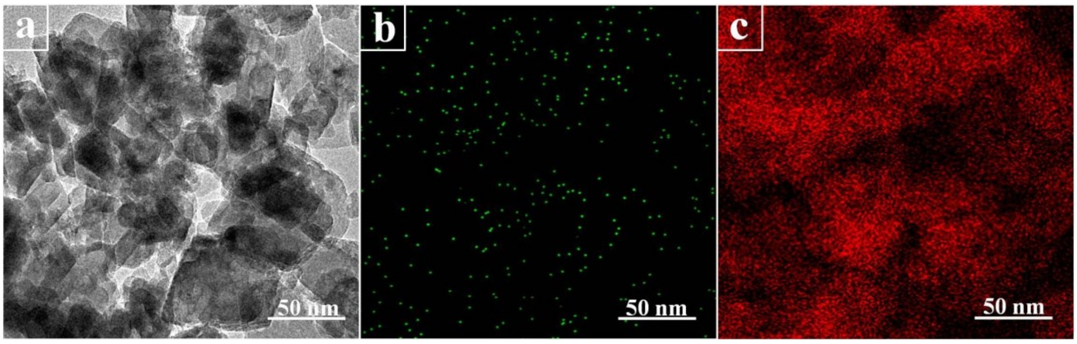

Figure S9. TEM images of the Au/Cu-MOF composites with 0.6 wt% Au determined by ICP-AES: (a) TEM image of Au/Cu-MOF with Au size of 0.8 nm; (b, c) STEM-EDX elemental mapping of Au/Cu-MOF with Au size of 0.8 nm, (b) represents Au elements and (c) represents Cu elements.

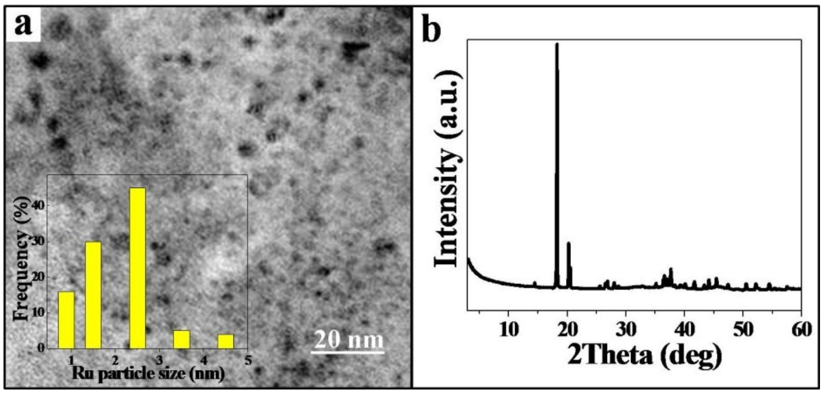

Figure S10. Characterizations of commercial Ru/C catalyst: (a) TEM image and inset is the particle size distribution; (b) XRD pattern.

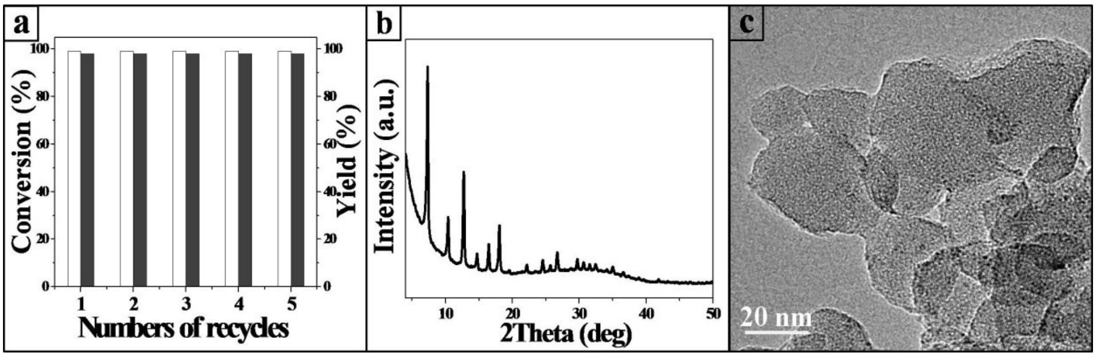

Figure S11. Reusability of the Ru/Zn-MOF catalyst at the reaction conditions of entry 1 of Table 2 (a); XRD pattern (b) and TEM image of Ru/Zn-MOF catalyst after used for five times.

Supplementary Tables

Table S1. The BET surface area (S), total pore volume (V) and average pore size (D) of different materials.

Entry	Samples	S ($m^2·g^{-1}$)	V ($cm^3·g^{-1}$)	D (nm)
1	Au/ Zn-MOF	849	0.792	22
2	Ru/ Zn-MOF	690	0.532	17
3	Pd/ Zn-MOF	706	0.496	16

References

S1 P. Zhang, J. Ma, X. C. Kang, H. Z. Liu, C. J. Chen, Z. R. Zhang, J. L. Zhang and B. X. Han, *Chem. Commun.,* 2017, **53,** 2162.

S2 A. L. Márquez, A. Medrano, L. A. Panizzolo and J. R. Wagner *J. Colloid Interface Sci.*, 2010, **341,** 101.

S3 Y. F. Yang, F. W. Wang, Q. H. Yang, Y. L. Hu, H. Yan, Y. Z. Chen, H. R. Liu, G. Q. Zhang, J. L. Lu, H. L. Jiang and H. X. Xu, *ACS Appl. Mater. Interfaces*, 2014, **6,** 18163.
